# Unraveling Fish Community Assembly Rules in Coastal China Seas Based on Hierarchical Modeling of Species Communities

**DOI:** 10.3390/ani15213108

**Published:** 2025-10-26

**Authors:** Li Lin, Yang Liu, Bin Kang

**Affiliations:** 1 Department of Animal Husbandry and Fisheries, Guizhou Vocational College of Agriculture, Guiyang 550000, China; danielin.fdu@icloud.com; 2 Key Laboratory of Mariculture (Ocean University of China), Ministry of Education, Qingdao 266003, China; 3 College of Ecological Engineering, Guizhou University of Engineering Science, Bijie 551700, China; yangliuouc1102@163.com

**Keywords:** community assembly, filtering rule, joint species distribution modeling, niche, species interaction

## Abstract

**Simple Summary:**

Coastal China seas’ fish communities face threats like overfishing and climate change, but how these communities react to these threats is unclear. This study aimed to understand what shapes these fish communities using a method of community modelling. We analyzed data on 384 fish species (1980–2018) and environmental factors. The results showed that temperature and salinity mostly determine fish distribution, and fish prefer silt over fine sand habitats. Goby fish have more connections with other fish. The findings help predict coastal fish communities and guide efforts to protect their biodiversity, benefiting ocean health and related human activities.

**Abstract:**

To address uncertainties in how threatened coastal China seas fish communities respond to stressors like overfishing and climate change, this study applied Hierarchical Modelling of Species Communities (HMSC) to disentangle the assembly rules shaping these communities, filling a critical gap in understanding their spatiotemporal dynamics. We analyzed data on 384 fish species (1980–2018) and key environmental factors, with variance partitioning revealing that environmental filtering dominated fish distributions (explaining over 99% of variance), far outweighing random effects (0.60%). Among environmental drivers, sea surface temperature (49.00%) and sea surface salinity (33.25%) were the most influential, while seafloor substrate and water depth played secondary roles; notably, fewer species occupied fine sand habitats, and more preferred silt habitats. Residual species associations—indicative of potential biotic interactions—were most frequent within Gobiidae, likely due to this highly diverse taxon’s specialized resource utilization and wide distribution, highlighting that biotic filtering is concentrated and ecologically relevant within this group. This work demonstrates HMSC’s utility in unraveling coastal fish community assembly, providing a robust basis for predicting community changes and guiding biodiversity conservation efforts that support ocean health and dependent human activities.

## 1. Introduction

Coastal habitats are essential for ecological processes and provide innumerable benefits and services to society and the entire planet as a whole [1]. These habitats, including soft shores, rocky shores and cliffs, hilly or flat coastal plains, narrow or wide coastal shelves, and a variety of wetlands, constitute various geomorphological features, weather regimes, and biomes, which support rich and indispensable biodiversity on planet earth [2]. The China seas include the Bohai Sea, the Yellow Sea, the East China Sea, and the South China Sea, which extend over 38 degrees of latitude and span from tropical to temperate climate zones, covering almost 5 million km^2^ and approximately 32,000 km of coastlines [3]. However, over the past half-century, the coastal ecosystems of the China seas have undergone severe degradation in fish community structure, driven by multiple anthropogenic and environmental stressors, including overfishing, marine pollution, biological invasions, and climate change [4,5]. China also has marine regions warming in the top 10% globally [4]. These pressures alter the structure and biodiversity of natural fish communities, thereby potentially affecting the functions and services that coastal ecosystems provide [6]. Therefore, there is an urgent need to understand how fish communities respond to changes in environmental conditions, as this knowledge is foundational to the formulation and prioritization of targeted, science-based conservation strategies.

Effective conservation management of the coastal China seas, where fish community structure has suffered severe deterioration over the past half century, requires knowledge of the rationales underlying how these specific coastal fish communities are assembled [7]. Studies exploring the correlations between communities and environmental conditions can be traced back to niche theory, which defines a species’ niche as a hypervolume, a multidimensional space bounded by the specific environmental characteristics (e.g., temperature, salinity, substrate type) that the species needs to survive, reproduce, and persist in a given habitat [8]. On the other hand, the neutral theory proposes that all individuals are ecologically identical and niche differences are not needed to explain community patterns, i.e., communities arise solely on random events, i.e., chance extinctions balanced by chance speciation [9]. In recent years, the importance of interspecific interactions to explain community structure has also been reinforced [10]. Therefore, not only environmental but also biotic factors and the stochastic nature of the community have been unified into the scope of community assembly rules framework, which incorporates the effects of species dispersal, environmental filtering, and biotic filtering [11]. First, dispersal defines the geographical distance at which a species can colonize [12]. Second, environmental filtering selects species according to their preference and tolerance to local abiotic conditions [13]. Third, species interact with each other through negative or positive biotic interactions [14].

Temperature has frequently been reported to be one of the most important factors driving marine fish community structure [15]. Other environmental factors, including salinity, depth, and water current, have been frequently proven influential at global or local scales [16,17,18]. Biotic filtering has also been proven to be influential for marine fish communities [19]. For instance, in the Pacific coral reefs near Papua New Guinea, in the Pacific coral reefs near Papua New Guinea, sympatric damselfishes *Dascyllus aruanus* and *D. melanurus* exhibit local-scale competitive interactions with *D. melanurus*, restricting the foraging range of *D. melanurus* to smaller prey in the presence of *D. aruanus* [20]. Trait-based approaches contribute to the understanding of community assembly [21]. For example, limiting similarity of fish functional traits is correlated with environmental filtering in coastal lagoon fish communities [22]. Fish functional traits and phylogenetic data can be used to better capture species’ responses to abiotic and biotic environments, as well as to improve the performance of community modeling by incorporating trait-phylogeny links to community assembly processes [23]. A plethora of fish resources investigations have been conducted in the coastal China seas tracing back to the 1980s [24,25,26]. These studies mostly focused on patterns of stock evaluation, community structure, or biodiversity in space and/or time, but insufficient efforts were dedicated to disentangling the community assembly rules shaping the spatiotemporal patterns of fish community structures.

Hierarchical Modelling of Species Communities (HMSC) is a multivariate hierarchical generalized linear mixed model designed for community ecology [23]. It has been built intrinsically in such a way that the model components, including fixed effects, random effects, species traits, and phylogenetic relationships, are conceptually related to community assembly processes [27]. Therefore, it is the optimal tool for a complex dataset that integrates occurrence data, multiple environmental variables, functional traits, and phylogenetic information [28]. Critically, these components are conceptually tied to the core community assembly processes, including environmental filtering, biotic filtering, and dispersal-related stochasticity, enabling us to parse their relative contributions to fish community structure in a system as geographically extensive and ecologically heterogeneous as the coastal China seas. With the increasing data availability of environmental variables and historical fish investigations in coastal China seas, this work applied HMSC to model the species responses to the gradients of environmental conditions, aiming to explore fish community assembly rules.

## 2. Materials and Methods

### 2.1. Study Area

**Figure 1:**
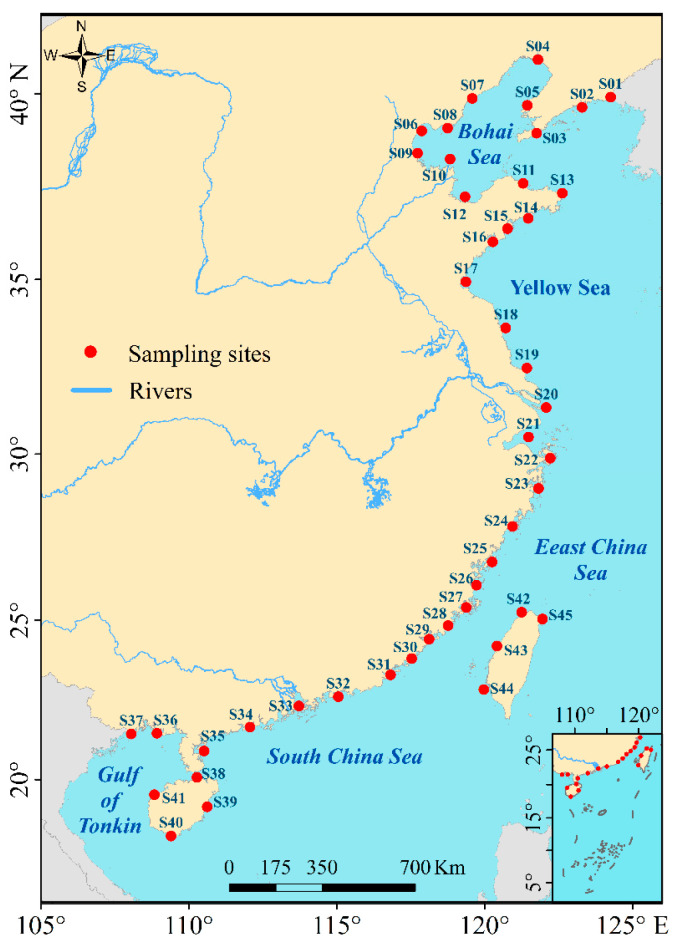
Study area and sampling sites [29]. The study area spans a longitudinal range of 105–125° E and a latitudinal range of 16–45° N. Fish survey data were collected at 45 sampling sites distributed along the coastal waters of the China seas encompassing the Bohai Sea, Yellow Sea, East China Sea, and South China Sea (with the Gulf of Tonkin included) over the period from 1980 to 2018. Detailed information on the sampling sites, including their respective codes, geographic coordinates, and additional relevant attributes, is presented in Appendix A—Table A1.

Fish investigation data were collected at 45 sampling sites distributed along the coastal waters of the China seas, encompassing four major marine regions: the Bohai Sea (latitude range: 37°07′–41°00′ N), the Yellow Sea (latitude range: 31°40′–37°07′ N), the East China Sea (latitude range: 21°54′–31°40′ N), and the South China Sea (latitude range: 16°00′–21°54′ N) (see Figure 1 for spatial distribution). Most observations were made in shallow waters below 40 m deep. Codes, names, and geographic coordinates of these sites are listed in Appendix A—Table A1.

### 2.2. Data Collection

#### 2.2.1. Species Occurrence Data Compilation

Data of 148 fish investigations in Chinese coastal waters between 1980 and 2018 at these 45 sites were compiled (see the data reference list in the files stated in Data Availability Statement). These investigations occurred independently and were hereinafter referred to as samples. Sampling methods were categorized in accordance with the definitions and classification criteria for fishing gear types established by the Food and Agriculture Organization of the United Nations (FAO) [30]. Most sampling methods were bottom trawls, including bottom otter trawls, bottom pair trawls, beam trawls, combined trawls, and other unspecified bottom trawls (Appendix B—Figure A1a). The sampling year was categorized based on the specific time period during which each fish survey was conducted (Appendix B—Figure A1b). Original fish taxonomic data were double-checked and validated with the records in FishBase to avoid invalid species, synonyms, and homonyms [31]. There were 384 valid fish species identified altogether, with the highest species richness of 250 at the Leizhou Bay (Appendix B—Figure A2a) and the highest species prevalence of 0.78 of the species *Konosirus punctatus* (Appendix B—Figure A2b).

#### 2.2.2. Environmental Data Compilation

Environmental variables included in this study (Appendix A—Table A2) were selected based on the following ecological rationales: (1) Water depth modulates benthic-pelagic coupling processes and shapes predator-prey interactions, which are critical to fish community structure [17]. (2) Seafloor substrate types directly influence the microhabitat suitability and resource availability for demersal fish species, thereby affecting their distribution and abundance [32]. (3) Water current directly affects temperature and food resources [18]. (4) water temperature, salinity, and pH directly affect fish physiology [16,33]. (5) Water net primary production exerts a fundamental regulatory influence on the structure of marine food webs, as it constitutes the primary energy input that supports the base of trophic hierarchies [34].

Data on mean water depth (depth) were retrieved from the data repository maintained by the National Oceanic and Atmospheric Administration (NOAA) [35]. Data on the types of seafloor surface substrate (substrate) were provided by the State Oceanic Administration of China, which adhered to the classification criteria outlined in the Shepard scheme [36,37]. Data on the northward component of the sea surface current (hereafter abbreviated as “current”), sea surface temperature (hereafter abbreviated as “SST”), and sea surface salinity (hereafter abbreviated as “SSS”) were obtained from the National Marine Data Center, which is part of the National Science & Technology Infrastructure Platform [38]. Data on sea surface water net primary production (NPP) and sea surface pH (pH) were acquired from the Copernicus Marine Environment Monitoring Service (CMEMS) [39]. While bottom temperature/salinity would be ideal, scarce, discontinuous historical data for the coastal China seas prevented their inclusion. Monthly datasets for SST, SSS, NPP, and pH were spatially and temporally averaged to match the specific month and year of each fish investigation. Present-day environmental variable layers were acquired from the Bio-ORACLE (Biological Ocean Atlas of Chemical and Physical Variables) database, a widely recognized repository for marine environmental data in ecological modeling [40]. Depth and substrate were treated as temporally invariant because the magnitude of temporal variation in these two variables was anticipated to be negligible at the same sampling site, and no additional historical datasets had been accessible to support temporal adjustments. Correlation coefficients among the selected environmental variables were all below 0.70 (Appendix B—Figure A3a), and the Variance Inflation Factor (VIF) values for each variable were less than a predefined threshold of 3 (Appendix B—Figure A3b), indicating the dataset exhibited an acceptable level of multicollinearity [41].

#### 2.2.3. Functional Trait Data Compilation

Functional traits were selected based on two criteria: (1) they are associated with different fish functions, including feeding habit, trophic level, swimming capability, habitat preference, and life history; (2) they tend to capture the response of fish species to abiotic and biotic environments, i.e., they are likely to be response traits [42]. We followed Brosse et al. [43] to derive nine morphological traits from their morphological measurements (Appendix B—Figure A4). These morphological measurements were recorded by ImageJ Version 1.53k [44]. Images were primarily sourced from regional fish atlases. Only adult specimens were photographed, with juveniles excluded to account for morphological variations associated with ontogeny. For species exhibiting sexual dimorphism, only male morphological traits were documented, as female specimens were rarely imaged across most species. Nine morphological traits (Appendix A—Table A3) were derived from these morphological measurements, including body elongation, body lateral shape, caudal peduncle throttling, oral gape position, pectoral fin vertical position, pectoral fin size, relative eye size, relative maxillary length and vertical eye position. For those fish with unusual morphologies, we followed Brosse et al. [43] and Villéger et al. [45] to apply the following rules: (1) for species lacking a visible caudal fin (e.g., Sternopygidae, Anguilidae, Plotosidae), the caudal peduncle throttling value was set to 1, with the assumption that caudal fin depth equals caudal peduncle depth; (2) for algivorous species with a subterminal mouth (e.g., Loricaridae, some Balitoridae), both oral gape position and relative maxillary length were assigned a value of 0; (3) for species without pectoral fins (e.g., some Synbranchiformes, Anguilliformes), the pectoral fin vertical position was set to 0; (4) for flatfishes, body depth was measured as body width, given their dorsoventrally flattened body and lateral resting posture. The ecological traits collected from FishBase were reviewed to ensure consistency with the morphological standard to confirm trait alignment.

We further followed the methodology of Trindade-Santos et al. [46] to extract eight additional biologically and ecologically relevant traits (Appendix A—Table A3) from the latest version of the FishBase database [31], including maximum lifespan, generation time, food consumption-to-biomass ratio, maximum body length, water column position, dietary habit, trophic level, and body shape. Given the unavoidable data gaps inherent in large-scale databases such as FishBase, a random forest algorithm was employed to impute missing values in the trait matrix (missing rate < 10%) [47]. Pearson correlation analyses were also conducted among these traits to verify their complementarity (Appendix B—Figure A5). The majority of these traits exhibited no significant correlation, confirming that they provided complementary information.

#### 2.2.4. Phylogenetic Data Compilation

Cytochrome oxidase I (COI) gene sequences were retrieved from the GenBank database [48]. Of the studied fish species, 355 (92.44%) had verified COI sequences, while no records were available for the remaining 29 species as of 1 July 2021. To improve the estimation of community-level phylogenetic information, surrogate sequences for these 29 species were sourced from their phylogenetically and morphologically close congeners (see species details in the Data Availability Statement). Sequences were aligned using Clustal W 2.0 [49]. The Akaike Information Criterion (AIC) was employed to select optimal parameters for constructing the Maximum Likelihood (ML) phylogeny [50]; the substitution model GTR + I + Γ best fit the empirical data based on AIC values (Appendix B—Figure A6), where “GTR” denotes the general time-reversible model, “+I” indicates optimization of the proportion of invariable sites, and “+Γ” represents optimization of the gamma rate parameter within the GTR framework. Finally, the ML phylogenetic tree under the GTR + I + Γ model was constructed using the “PhyML 3.1” executable program in R 4.1.1 [51].

### 2.3. Hierarchical Modelling of Species Communities

Data were fitted with Hierarchical Modelling of Species Communities (HMSC) [52], which includes a hierarchical layer asking how species respond to environmental covariates [27]. The phylogenetic tree and fish traits were included in the model to improve its performance [28]. Posterior distribution was sampled with four Markov Chain Monte Carlo (MCMC) chains, each of which was run for 300,000 iterations. The first 50,000 iterations were removed as burn-in. The chains were thinned by 1000 to yield 250 posterior samples per chain and so 1000 posterior samples in total. Three competing models were constructed for model validation as follows: (1) model_null, which included only an intercept and three random effects; (2) model_cov, which included an intercept and all predictors but excluded random effects; and (3) model_full, which included an intercept, all predictors, and three random effects. Five-fold cross-validation was performed [53]. Explanatory and predictive powers of these competing models were evaluated in terms of Area Under Curve (AUC) [54] and Tjur’s R^2^ (TjurR^2^) [55]. Explained variances were partitioned between fixed and random effects. One-way ANOVA with the method of Tukey’s “Honest Significant Difference” was applied to test if the average explained variances were significantly different between explaining variables at the confidence level of 0.95 [56]. The fine sand was used as the baseline category for the HMSC model’s dummy variable, i.e., substrate. The environmental niche of each species was quantified using the model’s estimated fixed-effect regression coefficients (β parameters) [52]. These β parameters directly describe the magnitude and direction of a species’ response to each environmental gradient, thereby defining its niche. More details for model fitting and model validation are specified in Appendix C.

## 3. Results

### 3.1. HMSC Model Validation

**Figure 2:**
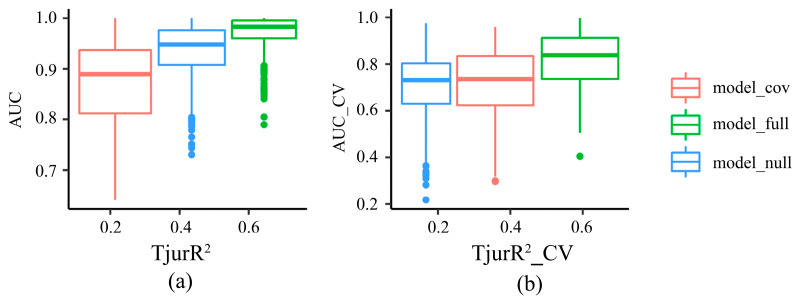
Validation of the competing models for Hierarchical Modelling of Species Communities (HMSC). The competing models include (1) model_null, which only includes an intercept and three random effects; (2) model_cov, which includes an intercept and all predictors, but no random effects; and (3) model_full, which includes an intercept, all predictors, and three random effects. AUC and TjurR^2^ in (**a**) indicate the index of Area Under Curve and Tjur’s R^2^ evaluated with the whole data set, respectively. AUC_CV and TjurR^2^_CV in (**b**) indicate the index of Area Under Curve and Tjur’s R^2^ with five-fold cross-validation, respectively.

The model_full, which incorporated both fixed and random effects, exhibited the highest AUC and TjurR^2^ values (Table 1, Figure 2), demonstrating superior explanatory and predictive power relative to the other competing models. In contrast, model_cov, which excluded all random effects, had lower explanatory and predictive power than model_full, indicating that spatial and temporal stochasticity contribute to explaining fish community patterns. Model_null, which included only an intercept, showed lower predictive ability compared with model_cov and model_full, confirming that explanatory variables are essential for interpreting community patterns. Additionally, AUC and TjurR^2^ values for each species in model_full were more convergent both pre- and post-cross-validation, indicating that this model generated the least uncertainty.

### 3.2. Fixed and Random Effects

**Figure 3:**
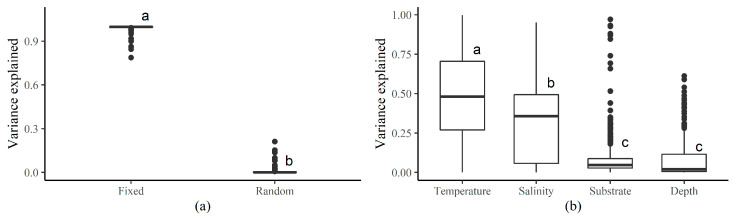
Variance partition for fixed and random effects (**a**), and for each explaining variable (**b**). Different lower-case letters on top of each box indicate significant differences between groups at the confidence level of 0.95.

Fixed effects of the HMSC model explained 99.40% of the distributions of coastal fish species on average, whereas random effects only explained 0.60% (Figure 3a), indicating strong assembly rules of environmental filtering. SST explained the majority of the variances (49.00%) of the species distributions, followed by SSS (33.25%), seafloor substrate (9.39%), and depth (7.76%) (Figure 3b), indicating that SST and SSS were the most influential drivers.

These results were corroborated by the variance partitioning for the distribution of each species (Appendix B—Figure A7). SST explained the majority of the variances for most species, followed by SSS, depth, substrate, and random effects, indicating that SST was the most important driver for the distribution of most species. Random effects explained only a few variances for most species, indicating that spatial and temporal stochasticity were limited across the range of fish species distributions.

### 3.3. Species Environmental Niche

**Figure 4:**
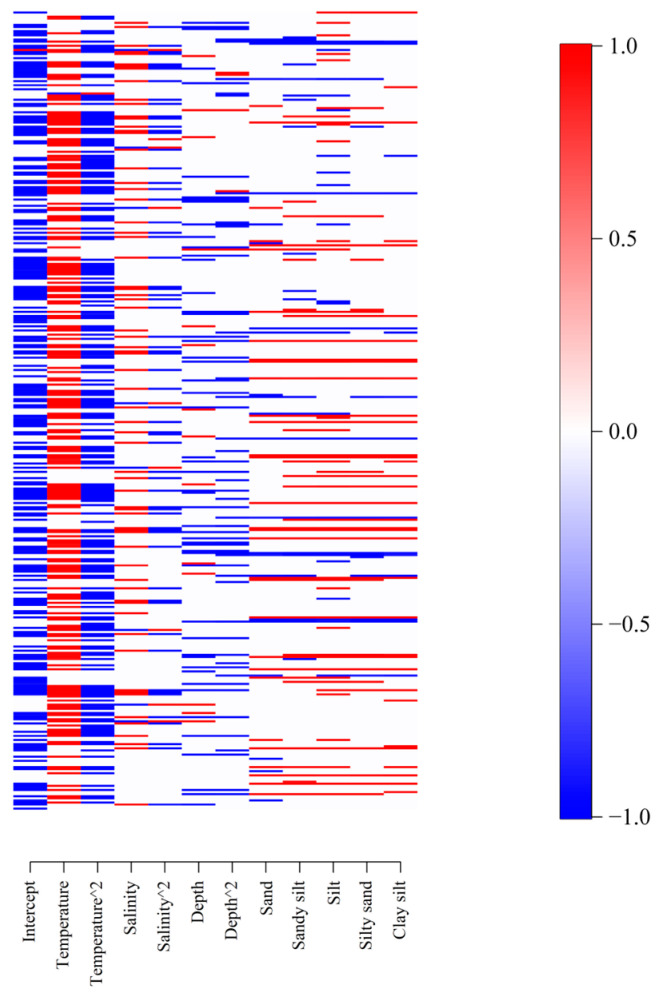
Heatmap of estimated parameters β, i.e., species niches. Gradient colors from blue to red show parameters that are estimated to be negative to positive, respectively, with at least 0.95 posterior probability. Symbol “^2” indicates the second-order term of the covariate, while the other counterpart without this symbol indicates the first order. Categorical variables are expanded to their levels. The first level is set as a baseline in the intercept of the model, and the other levels as dummy variables.

For most species, estimated intercepts were below 0 with at least 0.95 posterior probability (Figure 4), indicating that the expected distribution probability for most coastal fish species was smaller than 0.5, i.e., they were not widespread along the whole coastline. Estimated β parameters for the first-order term of SST for most species were below 0, and for the second-order term were higher than 0, indicating that there was an optimal SST value for most species. The mean estimated optimum SST value for these species clustered around 22 °C. The species with significant responses to SSS and depth were fewer than those with significant responses to SST, indicating that most species were more sensitive to SST than to SSS and depth. Estimated β parameters of different substrate categories for most species were positive, indicating that some species were less likely to be distributed in the baseline level of substrate, i.e., fine sand habitat.

**Figure 5:**
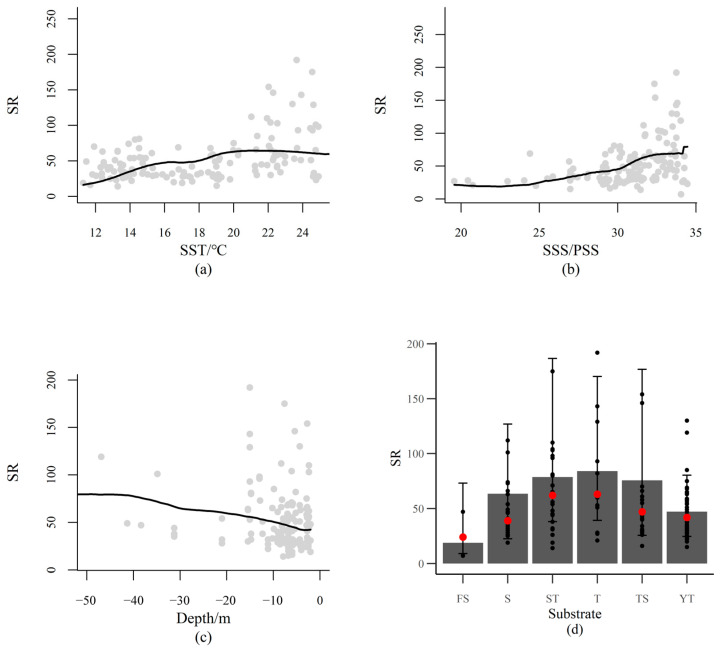
Marginal plots for the effects of environmental variables on species richness (SR). Marginal plots are visualized by plotting the response variable against one single explaining variable while maintaining other variables at their mean values. The panels show the expected values of SR along the gradients of sea surface temperature (SST, (**a**)), sea surface salinity (SSS, (**b**)), water depth (Depth, (**c**)), and substrate categories (Substrate, (**d**)). Red points in (**d**) indicate the median values.

Species richness (SR) increased with SST (Figure 5a), which was consistent with the GLMM for the preselection of explaining environmental variables. SR also increased with increasing SSS (Figure 5b), which indicated that most fish species preferred seawater with higher SSS. SR decreased when the water was shallower (Figure 5c), indicating SR was higher in deeper coastal waters. SR was lower in habitats with fine sand (FS), but higher in sandy silt (ST), silty (T), and silty sand (TS) habitats (Figure 5d), corroborating that most species preferred other substrate categories than fine sand habitats.

### 3.4. Residual Species Associations

**Figure 6:**
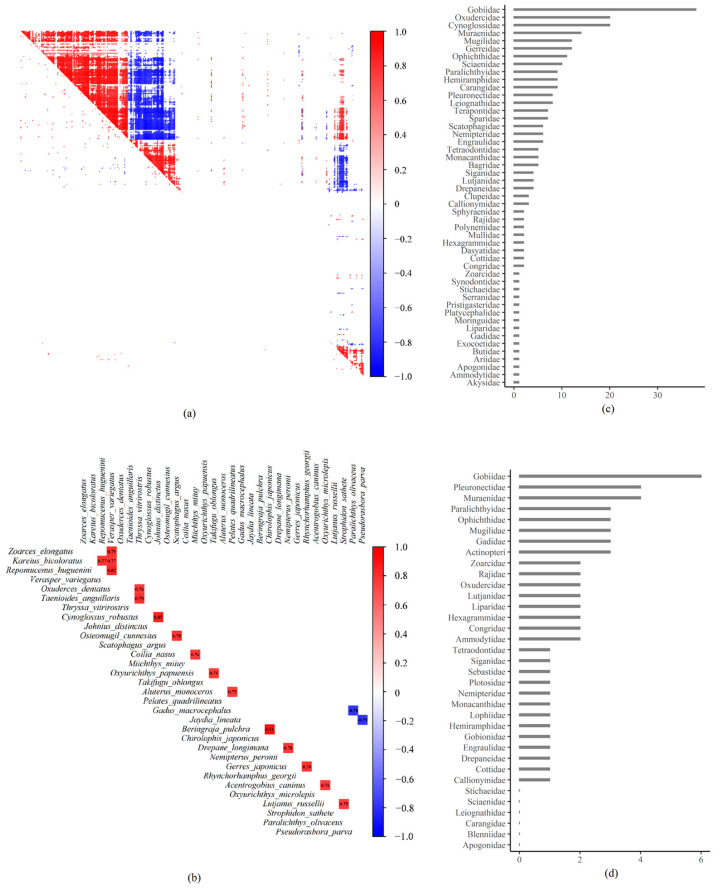
Positive and negative species associations. (**a**) Raw (upper right) and residual (lower left) species-to-species associations for those species pairs with at least 0.95 support probability for either a positive or negative association. (**b**) Top 10% positive or negative residual species associations with at least 0.95 support probability. (**c**) Basic statistics for positive residual associations within or between taxonomic families. (**d**) Basic statistics for negative residual associations within or between taxonomic families. Numbers in each colored square in (**a**,**b**) indicate coefficients of residual species associations. Horizontal axis in (**a**,**b**) indicates the counts of residual associations which occurs with at least one species within that family.

Both positive and negative co-occurrence patterns occurred in the fish communities (Figure 6). The percentage of species pairs that showed significant raw associations was 36.54%, while the percentage of species pairs that showed significant residual associations was 9.78%. Residual associations were fewer and more sporadic than raw associations, indicating that most species co-occurrences had been explained in model_full by the fixed and random effects (Figure 6a). The highest positive residual association occurred between mottled skate *Beringraja pulchra* and stichaeid fish *Chirolophis japonicus* with a value of 0.91, and the highest negative residual association occurred between stone moroko *Pseudorasbora parva* and cardinal fish *Jaydia lineata* with a value of −0.79 (Figure 6b). The most frequent residual associations occurred within or between *Gobiidae* species and other taxons with a percentage of 13.17% (Figure 6c,d).

## 4. Discussion

### 4.1. Performance of HMSC Modeling

Predictor selection is important for regression-based models to minimize the risk of over-parameterization and maximize the capability of extrapolation [57]. Anthropogenic disturbances, particularly marine pollution, overfishing, and management measures such as summer fishing moratoriums in the coastal seas of China, also exert specific impacts on coastal fish communities [4]. These factors are challenging to adequately incorporate into the current HMSC model, primarily due to the limited availability of valid historical data dating back to the 1980s. Consequently, the impacts of these unincluded factors are retained in the residuals or partially accounted for by the random effect of spatial autocorrelation. However, values of AUC were higher than 0.80 for model_full, which can be viewed as a “good” model according to Araujo et al. [58]. Tjur R^2^ for model_full was quite smaller compared with AUC, because for AUC the baseline that the model prediction is equally good as expected by random is 0.5, whereas for Tjur R^2^ the same baseline is 0 [52]. Values of Tjur R^2^ were higher than 0.35, which reassured that the model_full possessed reasonable explanatory and predictive power [55].

### 4.2. Species Niche and Environmental Filtering

Species thermal niche is one of the most popular subjects of environmental filtering. It is reported that temperature was the only influential factor for marine communities across 13 major taxa at global scales, including animals and plants [59]. In this work, both temperature and salinity were the most influential factors in coastal China seas, possibly because of the existence of many estuaries along the coastlines. China has the most abundant estuarine resources in the world [60]. Over twenty estuaries are characterized by an annual freshwater flux of 5 × 10^8^ m^3^, including the Yangtze River Estuary, Yellow River Estuary, and Pearl River Estuary. The estuary and its adjacent sea areas are a mixture of fresh and brackish water and therefore present a heterogeneous salinity gradient [61]. The vast gradient of temperature and salinity in the estuarine waters provides variate habitats for fish species and therefore influences the distributions of coastal fish species [15]. Species that adapt to brackish water or seek anadromous/catadromous migration compose different community structures in the estuary than in other salty waters [62]. Therefore, salinity was an important driver, especially for some freshwater/brackish species. For example, half-smooth tongue sole *Cynoglossus semilaevis*, cardinal fish *Jaydia lineata*, and silver pomfret *Pampus argenteus* are estuarine species, and salinity was the only significant driver for their distributions.

Water depth and seafloor substrate were also influential. Some fish adjust their bathymetrical behavior, such as vertical distribution in the water column and habitat selection, to cope with the thermal variation, which possibly correlates to the effect of depth on fish species distributions [63]. Seafloor substrate directly affects demersal habitat conditions. Different substrate types were categorized mainly by rugosity [36,37]. Responses of fish communities to rugosity were not congruent in different ecosystems. For example, in natural coral reefs, fish species richness and abundance were statistically higher in habitats with higher rugosity [64], but in rocky ecosystems, fish communities in substrate habitats with limestones were significantly different from those with granites [32]. There were scarce studies concerning the mechanism that why the species distributions in fine sand habitats were lower than in other habitats, but others have found that this is likely correlated to nursery and foraging habitat availability [32,65]. The availability of specific types of niches in habitats with specific rugosity types can lead to habitat specialization [66].

### 4.3. Species Associations and Biotic Filtering

It has been underlined that species co-occurrence patterns, i.e., species associations in HMSC, do not necessarily indicate species interspecific interactions [28]. Fish species associations that occurred in the coastal waters of China seas may be caused by a variety of factors, including but not limited to: (1) the environmental niches of different species are similar, and similar environmental filtering causes co-occurrence of species [67]; (2) there are positive or negative interspecific interactions between species, such as competition, parasitism, predation, etc. [68]; and (3) other random historical incidences, such as the formation of Taiwan Strait twenty thousand years ago in the last stage of the Paleolithic era that possibly indirectly influence fish species associations by shaping past habitat connectivity and geographic isolation because of the rising levels of the ocean [69]. However, the residual species associations are indicative of interspecific interactions, especially between those species that have never been studied [70]. For example, mottled skate *Beringraja pulchra* and stichaeid fish *Chirolophis japonicus* have a highly positive correlation (correlation coefficient 0.91). They are both small or medium-sized benthic fish feeding on small invertebrates [31]. Although there was no relevant report on the interspecific relationship between them, the significantly positive residual species association provided reasonable hypotheses that although they prey on the same food in the same area, the interspecific competition is weak, or even interspecific facilitation might exist. From the perspective of trophic niche breadth, these two species may differentiate in specific prey types. *Beringraja pulchra* possibly mainly targets burrowing invertebrates, and *Chirolophis japonicus* feed on epibenthic invertebrates, which reduces their resource overlap. Pacific cod *Gadus macrocephalus* is one of the important economic fish in northern coastal China [71]. It is a benthic carnivorous fish, and the adults feed on small fish [72]. Japanese flounder *Paralichthys olivaceus* is also a benthic fish, feeding on small fish and invertebrates [73]. The body size of *Gadus macrocephalus* and *Paralichthys olivaceus* is similar [31], and their negative residual association possibly suggests interspecific competition for food. *Gobiidae* is one of the largest families of marine fishes, with at least 2000 species described in 200 genera [74]. *Gobiidae* species, i.e., gobies, are generally benthic and small-sized (often less than 50 mm), and may occupy various niches in the substrate, including the bodies or burrows of invertebrates [75]. *Gobiidae* was also the most frequent taxon in this work (87 gobies out of 384 fish species). Therefore, more common resource utilization, segregation of habitat preferences, and food web correlations within or between gobies and other taxons were expected, which possibly explained why both positive and negative residual associatons were the most frequent for gobies. For instance, interspecific social interactions to a large extent explained the partial habitat separation between the three gobies under laboratory mud-sand and grass habitats [76]. Residual species association needs to cooperate with other ecological studies or laboratory experiments to further determine if interspecific interactions exist [77]. Nevertheless, residual species association provided us with additional useful information for diversity conservation or interspecific relationship research. For example, proper consideration of interspecific relationships of species can improve the performance of conservation prioritization.

Using static depth and substrate across 39 years is a limitation of the model due to data unavailability, especially given potential coastal sedimentation/erosion over decades. Future studies could integrate long-term data on anthropogenic disturbances (e.g., fishing intensity, marine pollution), depth, and substrate that were not fully accounted for in this work into the HMSC framework, and combine field surveys or laboratory experiments to verify the potential interspecific interactions of *Gobiidae* and other taxa, thereby further refining the prediction of coastal fish community dynamics and providing more precise guidance for marine biodiversity conservation.

## 5. Conclusions

The Hierarchical Modelling of Species Communities (HMSC) employed in this study exhibited strong explanatory and predictive power. Environmental filtering drives the spatiotemporal patterns of fish communities in coastal seas. The influence of random effects is limited. Besides SST, other variables, including SSS, seafloor substrate, and water depth, are also significantly influential factors driving coastal fish communities. More interspecific associations occur for Gobiidae species, possibly because of their wide range of distribution and resource utilization. This work provides important information for coastal fish conservation management and is precursory for predictions of coastal fish communities.

## Figures and Tables

**Table 1:** Mean and standard deviation values of the index of Area Under Curve and Tjur’s R2 of different models.

	AUC *	AUC_CV	TjurR^2^	TjurR^2^_CV
model_cov	0.87 ± 0.08	0.72 ± 0.14	0.26 ± 0.14	0.15 ± 0.14
model_null	0.93 ± 0.6	0.71 ± 0.13	0.13 ± 0.04	0.07 ± 0.08
model_full	0.97 ± 0.04	0.82 ± 0.12	0.49 ± 0.13	0.36 ± 0.13

* The competing models include (1) model_null, which only includes an intercept and three random effects; (2) model_cov, which includes an intercept and all predictors, but no random effects; and (3) model_full, which includes an intercept, all predictors, and three random effects. AUC and TjurR^2^ indicate the index of Area Under Curve and Tjur’s R^2^ evaluated with the whole data set, respectively. AUC_CV and TjurR^2^_CV indicate the index of Area Under Curve and Tjur’s R^2^ with five-fold cross-validation, respectively.

**Figure A1:**
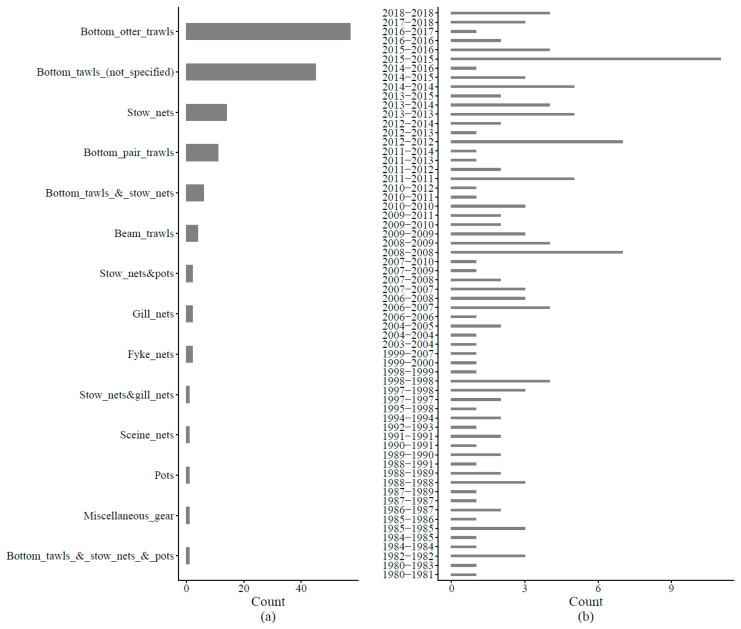
Basic statistics for the sampling years and sampling methods. Sampling methods (**a**) are identified according to the definition and classification of fishing gear categories by Food and Agriculture Organization of the United Nations (FAO) [30]. The sampling year (**b**) indicates the period when the fish investigation was conducted. Length of the horizontal column representing “2018–2018” indicates the number of investigations occurred in that year.

**Figure A2:**
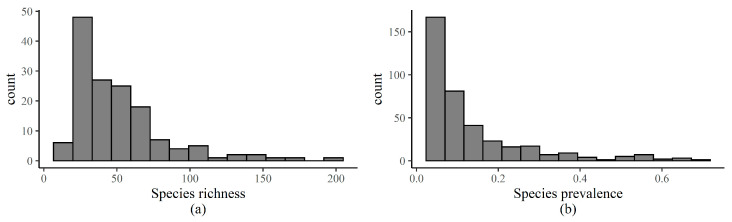
Histograms showing the distribution of (**a**) species richness (number of species) and (**b**) species prevalence (frequency of species occurrence) across samples. The *x*-axis in (**a**) represents species richness, and in (**b**) represents species prevalence; the *y*-axis in both panels represents the count of samples falling into each bin.

**Figure A3:**
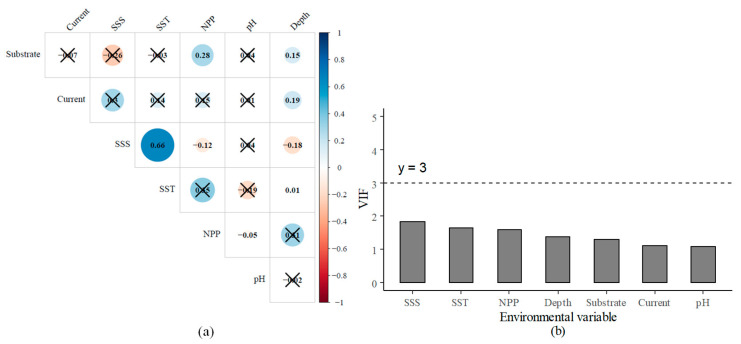
Multicollinearity analysis of environmental variables. (**a**) Correlation matrix heatmap depicting the Pearson correlation coefficients between environmental variables, including the northward component of the sea surface current (Current), sea surface salinity (SSS), Sea surface temperature (SST), net primary production (NPP), and sea surface pH (pH). Cross marks indicate non-significant correlations, while colored cells represent significant correlations (blue for positive, red for negative) with corresponding coefficient values. (**b**) Variance Inflation Factor (VIF) bar plot showing the VIF values for each environmental variable; a dashed line at y = 3 serves as a threshold for identifying potential multicollinearity.

**Figure A4:**
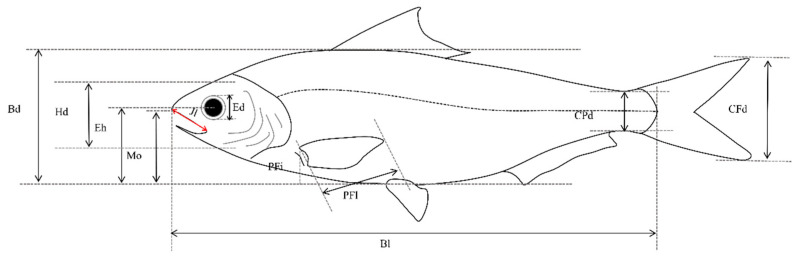
Morphological measurements of the fish. The measurements include standard length (Bl), body depth (Bd), head depth (Hd), caudal peduncle depth (CPd), eye diameter (Ed), eye position (Eh), mouth height (Mo), maxillary jaw length (Jl), pectoral fin length (PFl), and pectoral fin position (PFi).

**Figure A5:**
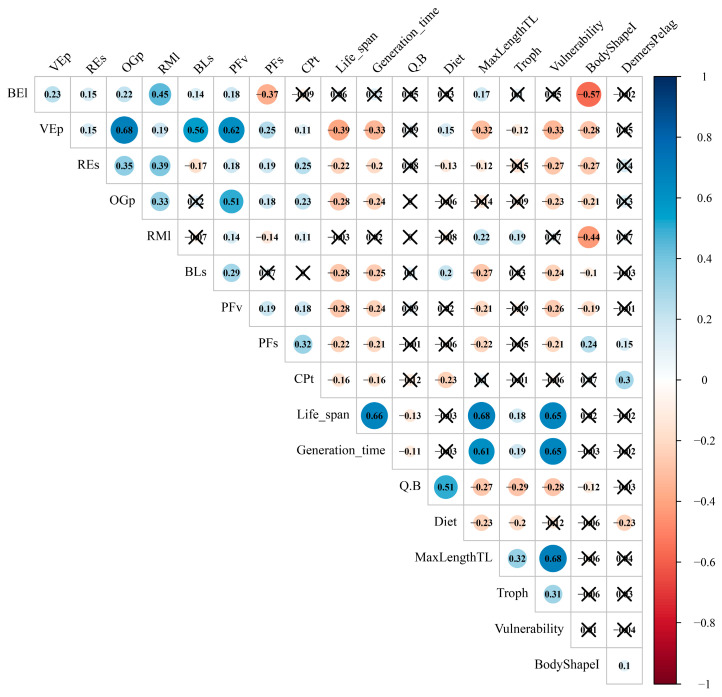
Correlation matrix heatmap depicting the Pearson correlation coefficients between functional traits. Cross marks indicate non-significant correlations, while colored cells represent significant correlations (blue for positive, red for negative) with corresponding coefficient values.

**Figure A6:**
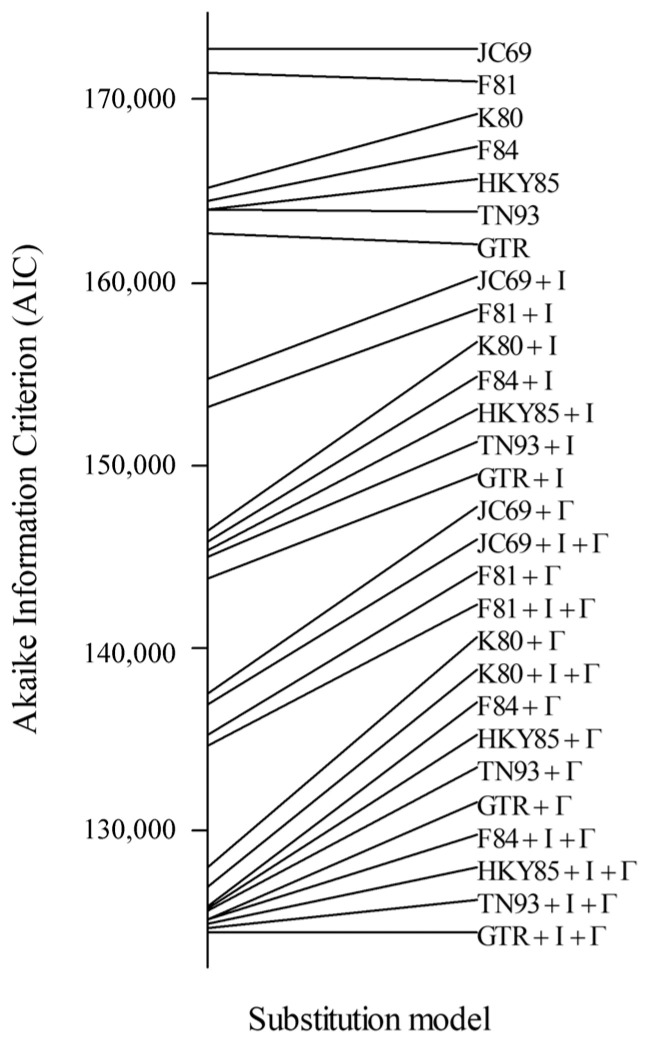
Akaike Information Criterion (AIC) for Maximum Likelihood (ML) phylogenetic analysis. The substitution model “GTR + I + Γ” is the one that achieves the lowest AIC, where “GTR” denotes the general time-reversible model; meanwhile, “+I” and “+G” indicate that invariant sites and a gamma distribution of substitution rates have been specified.

**Figure A7:**
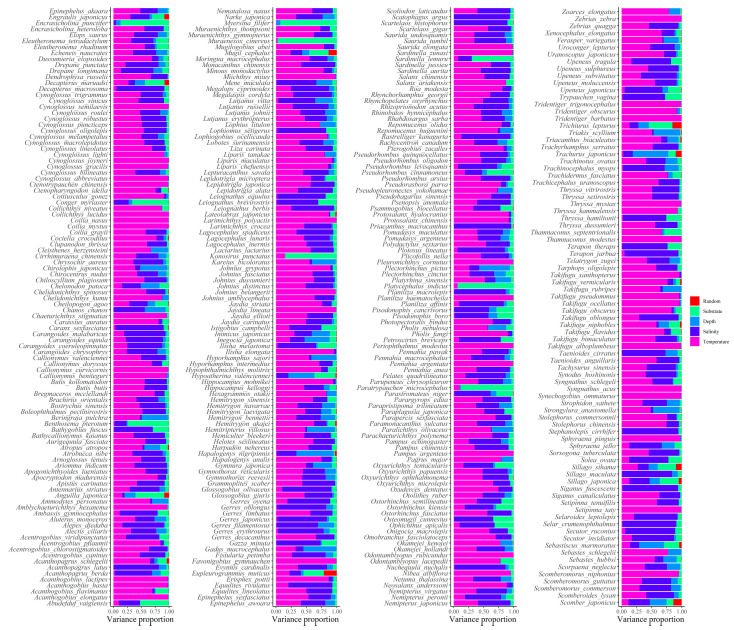
Variance partitioning for the distributions of each species.

**Figure A8:**
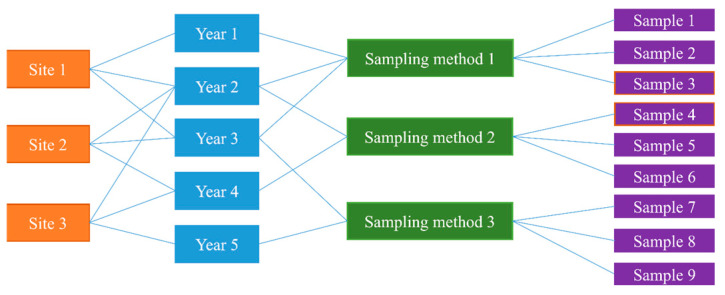
Demonstration of the data structure. Sample 1, 2, 3 etc. represent different fish investigations. There are altogether 45 sites, 14 levels of sampling methods, 62 levels of sampling years, and 148 samples.

**Figure A9:**
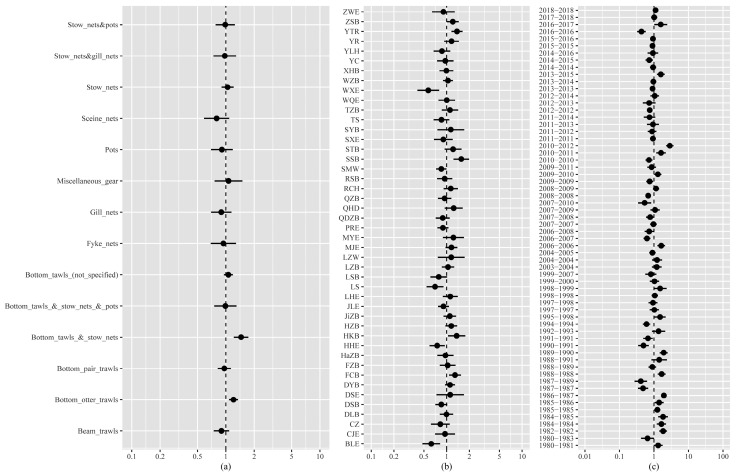
Influence of random effects on fish species richness. The *X*-axis indicates the standardized intercept of the generalized mixed model fitting species richness, and the *Y*-axis indicates different categories of (**a**) sampling method, (**b**) sampling site, and (**c**) sampling year. Categories of sampling method are identified according to the definition and classification of fishing gear categories by Food and Agriculture Organization of the United Nations (FAO). Codes of sampling sites are listed in Appendix A—Table A1. The sampling year indicates the period when the fish investigation was conducted. For example, 2017–2018 means the investigation was carried out from 2017 to 2018.

**Figure A10:**
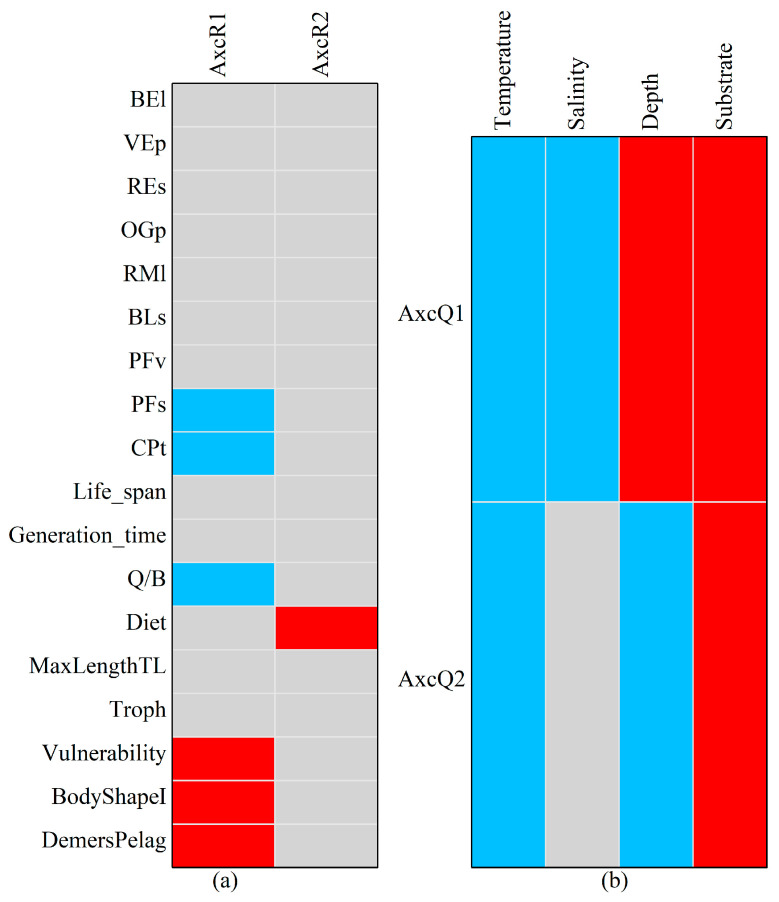
Correlations between fish traits and environmental variables based on fourth-corner combined with RLQ analysis. (**a**) Fourth-corner tests between the first two RLQ axes for environmental gradients (AxR1/AxR2) and traits. (**b**) Fourth-corner tests between the first two RLQ axes for trait syndromes (AxQ1 and AxQ2) and environmental variables. Positive significant associations are represented by red lines and cells, and negative significant associations by blue lines and cells. Variables with no significant associations are shown in grey. *p* values were adjusted for multiple comparisons using the FDR procedure. Environmental variables include sea surface salinity (SSS), sea surface temperature (SST), net primary production (NPP), mean depth, the north component of sea surface current (current), and sea surface pH (pH). Codes for traits are explained in Appendix A—Table A3.

**Figure A11:**
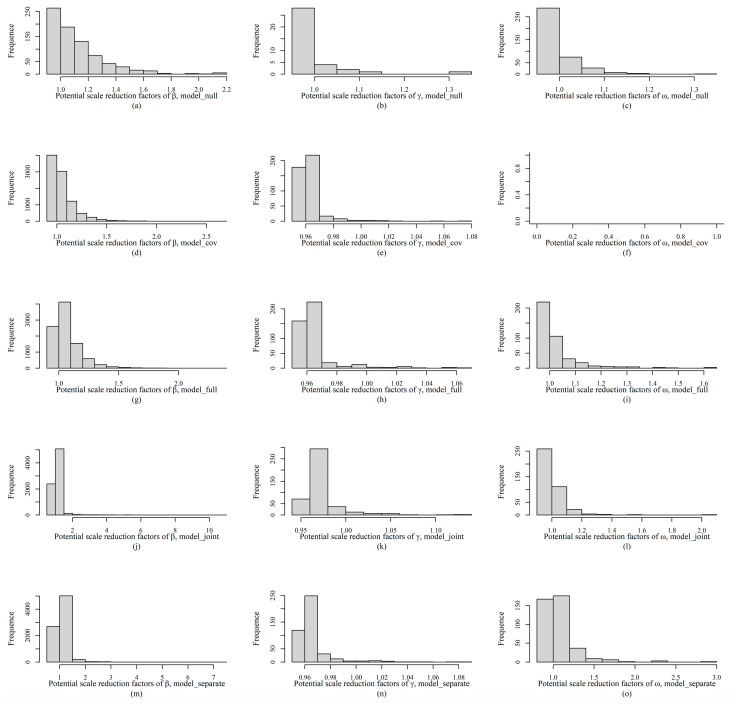
MCMC convergence diagnostics for the competing models in terms of potential scale reduction factors. Potential scale reduction factors are shown for species niches, i.e., coefficients of the explaining environmental variables (β), influences of traits on niches (γ), and residual species associations (ω). The competing models include (1) model_null (**a**–**c**) which only included random effects; (2) model_cov (**d**–**f**) which include all predictors, but no random effects; (3) model_full (**g**–**i**) which included all predictors and random effects; (4) model_joint (**j**–**l**) which performed a variable selection jointly for all species by a spike and slab prior process; and (5) model_separate (**m**–**o**) which performed a variable selection individually for each species also by the method of spike and slab prior, but species selected these variables separately.

## Data Availability

The data underlying this study are openly available in Figshare at https://doi.org/10.6084/m9.figshare.21864348.v3 [78].

## Abbreviations

The following abbreviations are used in this manuscript:

HMSC	Hierarchical Modelling of Species Communities
SST	Sea surface temperature
SSS	Sea surface salinity
NPP	Sea surface water net primary production
COI	Cytochrome oxidase I
AIC	Akaike Information Criterion
ML	Maximum Likelihood
MCMC	Markov Chain Monte Carlo chains
AUC	Area Under Curve
TjurR^2^	Tjur’s R^2^
SR	Species richness
FS	fine sand
ST	sandy silt
T	silty
TS	silty sand

## Appendix A. Tables

**Table A1:** Sampling site information.

Site Code	Name	Longtitue	Latitude	Type
YLH	Yalu_River_Estuary	124.2700	39.9099	estuary
QDZB	Qingduizi_Bay	123.3137	39.7403	bay
DLB	Dalian_Bay	121.7656	38.9624	bay
LHE	Liaohe_Estuary	121.8120	40.8753	estuary
FZB	Fuzhou_Bay	121.4484	39.6933	bay
HHE	Haihe_Estuary	117.8812	39.0222	estuary
QHD	Qinhuangdao	119.5885	39.8748	bay
TS	Tangshan	118.7555	39.0972	bay
CZ	Cangzhou	117.7445	38.4324	bay
YR	Yellow_River_Estuary	119.2836	37.8584	estuary
TZB	Taozi_Bay	121.3100	37.6280	bay
LZB	Laizhou_Bay	119.3451	37.6836	bay
RCH	Rongcheng_Bay	122.6361	37.3640	bay
RSB	Rushan_Bay	121.4822	36.6802	bay
LSB	Laoshan_Bay	120.7858	36.4008	bay
JiZB	Jiaozhou_Bay	120.2879	36.0395	bay
HZB	Haizhou_Bay	119.3881	34.8817	bay
YC	Yancheng	120.8215	33.4082	bay
LS	Lvsi_Fishang_Ground	121.4388	32.4997	bay
YTR	Yangtze_River_Estuary	121.9999	31.4612	estuary
HaZB	Hangzhou_Bay	121.4922	30.4973	bay
ZSB	Zhoushan_Bay	122.2268	29.8811	bay
SMW	Sanmen_Bay	121.4675	29.0681	bay
WZB	Wenzhou_Bay	121.0176	27.9848	bay
SSB	Sansha_Bay	119.8136	26.5192	bay
MJE	Min_River_Estuary	119.6757	26.0894	estuary
XHB	Xinghua_Bay	119.3879	25.3941	bay
QZB	Quanzhou_Bay	118.7726	24.8341	bay
JLE	Jiulongjiang_Esturay	117.9927	24.4342	estuary
DSB	Dongshan_Bay	117.5439	23.8171	bay
STB	Shantou_Bay	116.8310	23.3084	Bay
DYB	Daya_Bay	114.6685	23.7069	bay
PRE	Pearl_River_Estuary	113.7554	22.5379	estuary
MYE	Moyang_Estuary	112.0765	21.7131	estuary
LZW	Leizhou_Bay	110.5258	20.9146	bay
FCB	Fangchenggang_Bay	108.3700	21.5411	bay
BLE	Beilunhe_Estuary	108.0638	21.4557	estuary
HKB	Haikou_Bay	110.2825	20.0773	bay
WQE	Wanquan_Estuary	110.6023	19.1566	estuary
SYB	Sanya_Bay	109.4807	18.2711	bay
CJE	Changjiang_Estuary	108.9337	19.5213	bay
DSE	Danshuihe_Estuary	121.4108	25.1792	estuary
WXE	Wuxi_Estuary	120.4760	24.2046	estuary
ZWE	Zengwenxi_Estuary	121.4108	25.1792	estuary
SXE	Shuangxi_Estuary	121.9652	25.0380	estuary

**Table A2:** Environmental variables that potentially affect fish communities in coastal China seas.

Variable	Description	Unit/Levels
Depth	Mean water depth.	m
Substrate	Types of seafloor surface substrate.	Fine sand (FS), sand (S), sandy silt (ST), silt (S), silty sand (TS), and clay silt (YT)
Current	The north component of sea surface current.	m/h
SST	Sea surface temperature.	°C
SSS	Sea surface water salinity.	PSS
pH	Sea surface water pH reported on total scale.	1
NPP	Sea surface net primary production of biomass expressed as carbon per unit volume in sea water.	mg/m^3^/day

**Table A3:** Functional traits which are potentially responsive to environmental variables. Source indicates how the functional traits are compiled. The first nine morphological traits are derived from the morphological measurements. These measurements are standard length (Bl), body depth (Bd), Head depth (Hd), Caudal peduncle depth (CPd), eye diameter (Ed), eye position (Eh), mouth height (Mo), maxillary jaw length (Jl), pectoral fin length (PFl), and pectoral fin position (PFi). For example, Bl/Bd stands for Bl divided by BD. The last nine traits are obtained from FishBase.

Trait	Code	Source	Group	Type
Body elongation	BEl	Bl/Bd	Morphological	Continuous
Vertical eye position	VEp	Eh/Bd	Morphological	Continuous
Relative eye size	REs	Ed/Hd	Morphological	Continuous
Oral gape position	OGp	Mo/Bd	Morphological	Continuous
Relative maxillary length	RMl	Jl/Hd	Morphological	Continuous
Body lateral shape	BLs	Hd/Bd	Morphological	Continuous
Pectoral fin vertical position	PFv	PFi/Bd	Morphological	Continuous
Pectoral fin size	PFs	PFl/Bl	Morphological	Continuous
Caudal peduncle throttling	CPt	CFd/CPd	Morphological	Continuous
Body shape	BodyShape	FishBase	Morphological	Categorical
Maximum life span	Life_span	FishBase	Life History	Continuous
Generation time	Generation_time	FishBase	Life History	Continuous
Food consumption to biomass ratio	Q.B	FishBase	Life History	Continuous
Maximum body length	MaxLengthTL	FishBase	Life History	Continuous
Position in water column	DemersPelagI	FishBase	Ecological	Categorical
Diet	Diet	FishBase	Ecological	Categorical
Trophic level	Troph	FishBase	Ecological	Continuous

## Appendix C. Procedures for HMSC

### Appendix C.1. Preselection of Random Effects

Possible random effects were checked and determined in advance by fitting species richness (SR) in a generalized linear mixed model (GLMM) with log link and Poisson error distribution [79,80]. Therefore, three crossed random effects were initially considered according to the data structure (Figure A8) [81], including (1) random intercept of sampling site, to account for spatial non-independence of the samples within sites; (2) random intercept of sampling year, to account for temporal non-dependence of the samples; and (3) random intercept of sampling method, to account for the variance caused by different sampling methods. GLMM was fitted with different combinations of random effects and Akaike information criterion (AIC) was evaluated [82]. The random effect combination which creates the lowest AIC value was random intercepts of sampling site plus sampling year (Table A4). This was corroborated by the influence of random effects on the intercept of GLMM (Figure A9). Values of the intercepts under different sampling methods did not deviate from the expected value of 1 (Figure A9a), but sampling sites and sampling years influenced some values of the intercepts Figure A9b,c), indicating that different sampling methods in the data did not significantly influence the expected values of SR. Therefore, random intercepts of sampling site and sampling year were used in subsequent HMSC models. To account for potential spatial autocorrelation, another random effect of spatial autocorrelation based on geographic coordinates of the sampling sites was also included.

**Table A4:** Values of the Akaike information criterion (AIC) of the models with different combinations of random effects. No random effect indicates the regression model only includes fixed effects. Site, method, and year represent the random intercept of sampling site, sampling year, and sampling method, respectively. The plus sign “+” indicates the inclusion of different random effects simultaneously, e.g., site + method means the component model includes both random intercepts of sampling site and sampling method.

Combination	df	AIC
No random effect	12	2315.83
Site	13	1892.60
Method	13	2081.80
Year	13	1490.26
Site + method	14	1764.38
Site + year	14	1382.03
Method + year	14	1424.43
Site + method + year	15	1365.84

### Appendix C.2. Preselection of Explaining Variables and Fish Traits

In case of over-parameterization by environmental variables, the significance of environmental variables was checked in the GLMM fitting SR with random sampling site and sampling year. Sea surface temperature (SST), sea surface salinity (SSS), depth, and substrate were those variables that significantly influenced the patterns of SR (Table A5). In case of over-parameterization by fish functional traits, a fourth-corner combined with RLQ analysis was performed [83]. The functional traits which significantly correlated with the environment variables (Figure A10) were pectoral fin size (PFs), caudal peduncle throttling (CPt), food consumption to biomass ratio (Q/B), body shape (BodyShapeI), and position in the water column (DemersPelag). Therefore, these environmental variables and functional traits were used in subsequent HMSC models. We included both the first-order and second-order terms of SST, SSS, and depth, assuming that most species had their optimum preferences for these variables [16,84,85].

**Table A5:** Significance of different environmental variables. Explaining variables are net primary production (NPP), sea surface pH (pH), sea surface salinity (SSS), sea surface temperature (SST), the north component of sea surface current (current), mean water depth (depth), and types of seafloor surface substrate (substrate). The categories of the substrate are fine sand (FS), sand (S), sandy silt (ST), silt (S), silty sand (TS), and clay silt (YT). FS is treated as a basic level in the model and integrated into the intercept. With the multiplicative Poisson model, incidence rate ratios are the exponents of the regression coefficients, CI refers to the confidence interval at the level of 95%.

Predictors	Incidence Rate Ratios	CI	*p*	
(Intercept)	23.12	10.60–50.42	<0.001	***
Substrate [S]	2.35	1.03–5.37	0.043	*
Substrate [ST]	2.23	1.02–4.87	0.045	*
Substrate [T]	2.34	0.99–5.56	0.053	
Substrate [TS]	1.69	0.73–3.88	0.219	
Substrate [YT]	1.98	0.89–4.39	0.094	
SST	1.23	1.10–1.37	<0.001	***
SSS	1.16	1.02–1.31	0.019	**
Depth	1.16	1.02–1.33	0.023	*
NPP	0.82	0.72–0.94	0.065	
pH	0.92	0.85–1.00	0.064	
Current	1.04	0.98–1.11	0.155	

Significance codes: *** *p* < 0.001, ** *p* < 0.01, * *p* < 0.05.

### Appendix C.3. HMSC Model Fitting and Validation

Data were fitted with Hierarchical Modelling of Species Communities (HMSC) [27,52,86]. Bayesian generalized linear mixed model with a probit link and binomial error distribution was applied in HMSC models with the R-package Hmsc assuming the default prior distributions [84]. Posterior distribution was sampled with four Markov Chain Monte Carlo (MCMC) chains, each of which was run for 300,000 iterations, of which the first 50,000 were removed as burn-in. The chains were thinned by 1000 to yield 250 posterior samples per chain and so 1000 posterior samples in total. MCMC convergence was checked by examining the potential scale reduction factors of the model parameters [85]. Three competing models were constructed for model validation, including: (1) model_null which only included an intercept and three random effects; (2) model_cov which include an intercept and all predictors, but no random effects; and (3) model_full which included an intercept, all predictors, and three random effects. MCMC chain convergence, which was evaluated in terms of potential scale reduction factors [86], was acceptable for these competing models as the potential scale reduction factors for parameters β, γ, and ω were essentially converged to one (Figure A11).

Explanatory and predictive powers of these competing models were evaluated in terms of Area Under Curve (AUC) [54] and Tjur’s R^2^ (TjurR^2^) [55]. To compute explanatory power, model predictions were projected based on the whole data set. To compute predictive power, a five-fold cross-validation was performed, in which the sampling units were assigned randomly to five folds, and predictions for each fold were based on a model fitted to the remaining four folds [86]. Therefore, AUC and TjurR^2^ were calculated based on all data sets, indicating the explanatory power of the model, while AUC_CV and TjurR^2^_CV were based on a five-fold cross-validation, indicating predictive power.
