# Correction: Lin et al. Unraveling Fish Community Assembly Rules in Coastal China Seas Based on Hierarchical Modeling of Species Communities. *Animals* 2025, *15*, 3108

**DOI:** 10.3390/ani16040554

**Published:** 2026-02-11

**Authors:** Li Lin, Yang Liu, Bin Kang

**Affiliations:** 1 Department of Animal Husbandry and Fisheries, Guizhou Vocational College of Agriculture, Guiyang 550000, China; danielin.fdu@icloud.com; 2 Key Laboratory of Mariculture (Ocean University of China), Ministry of Education, Qingdao 266003, China; 3 College of Ecological Engineering, Guizhou University of Engineering Science, Bijie 551700, China; yangliuouc1102@163.com

**Missing Funding**

In the original publication [1], the funder “Kaiyang Huamaya Wild Bee Farm” was not included. The authors state that the scientific conclusions are unaffected. This correction was approved by the Academic Editor. The original publication has also been updated.
